# Effects of Two Invasive Weeds on Arthropod Community Structure on the Central Plateau of New Zealand

**DOI:** 10.3390/plants9070919

**Published:** 2020-07-20

**Authors:** Evans Effah, D. Paul Barrett, Paul G. Peterson, Murray A. Potter, Jarmo K. Holopainen, Andrea Clavijo McCormick

**Affiliations:** 1 School of Agriculture and Environment, Massey University, Tennent Drive, Palmerston North 4410, New Zealand; E.Effah@massey.ac.nz (E.E.); D.P.Barrett@massey.ac.nz (D.P.B.); M.Potter@massey.ac.nz (M.A.P.); 2 Manaaki Whenua—Landcare Research, Riddet Road, Massey University, Palmerston North 4410, New Zealand; Petersonp@landcareresearch.co.nz; 3 Department of Environmental and Biological Sciences, University of Eastern Finland, Yliopistonranta 1 E, FI-70210 Kuopio, Finland; jarmo.holopainen@uef.fi

**Keywords:** exotic weeds, invasion ecology, invasive species, plant community composition, arthropod diversity, arthropod community composition

## Abstract

Heather (*Calluna vulgaris*) and broom (*Cytisus scoparius*), originally from Europe, are the main invasive plants on New Zealand’s North Island Central Plateau, where they threaten native flora and fauna. Given the strong link between arthropod communities and plants, we explored the impact of these invasive weeds on the diversity and composition of associated arthropod assemblages in this area. The arthropods in heather-invaded areas, broom-invaded areas, and areas dominated by the native species mānuka (*Leptospermum scoparium*) and *Dracohyllum* (*Dracophyllum subulatum*) were collected and identified to order. During summer and autumn, arthropods were collected using beating trays, flight intercept traps and pitfall traps. Diversity indices (Richness, Shannon’s index and Simpson’s index) were calculated at the order level, and permutational multivariate analysis (PERMANOVA) was used to explore differences in order-level community composition. Our results show a significant variation in community composition for all trapping methods in both seasons, whereas invasive plants did not profoundly impact arthropod order richness. The presence of broom increased arthropod abundance, while heather was linked to a reduction. Under all possible plant pairings between heather, broom, mānuka, and *Dracophylum*, the impact of neighbouring plant identity on arthropod community composition was further explored for the samples collected using beating trays. The results suggest that during plant invasion, arthropod communities are affected by neighbouring plant identity and that impacts vary between arthropod sampling methods and seasons.

## 1. Introduction

Increased human migration, trade, and climate change are significant factors contributing to the spread of plants beyond their natural boundaries [1,2,3]. Some introduced plants survive, spread, and become invasive in new habitats. A variety of factors contribute to the success of invasive plants in their new environment, including biogeographic affinity between their native and invasive range, rapid and high reproductive outputs [4], rapid growth and high-stress tolerance [5,6], lack of specialist natural enemies [7], high phenotypic plasticity [8,9,10], the ability to release phytotoxic compounds into the environment [11], and the potential to rob native plants of their mutualists [12]. The threats posed by exotic invasive plants have gained much attention in recent years, with loss of biodiversity often associated with plant invasion [13,14].

Invasive plants change the vegetation structure and composition of their new habitats through direct competition or modification of the environment [15]. Arthropod communities are vulnerable to these changes due to the impact of microclimatic factors on their development and their close interaction with plants. Several studies report a significant decrease in arthropod diversity and abundance in response to plant invasions, as reviewed by Litt and colleagues [15], and others suggest that arthropod assemblages could be restored when invasive plants are eradicated [16,17,18]. However, arthropods fill diverse niches and ecological roles, and their responses to plant invasion may vary. For instance, some invasive plants may attract pollinators [19], provide alternative resources for generalist herbivores, or create favourable conditions for predators and decomposers [15]. It is therefore important to explore changes in arthropod community composition in different invasion scenarios through the seasons, using a range of sampling techniques to avoid faulty generalisation.

In New Zealand, Tongariro National Park lies within the Central North Island’s Volcanic Plateau, an area originally covered by subalpine shrubland, tussockland, and montane *Nothofagus* and *Libocedrus* forests. Volcanic activity and forest loss due to burning have created large areas of tussockland, where only a few woody perennials like *Dracophyllum* (*Dracophyllum subulatum*) and mānuka (*Leptospermum scoparium*) persist [20]. However, this ecosystem is threatened by the spread of exotic invasive weeds including heather (*Calluna vulgaris*) and broom (*Cytisus scoparius*), both of European origin. Heather was deliberately planted in the Tongariro National Park by European settlers in 1912 [21] and is now the most widespread weed in the area, while the broom invasion began in the 1960s [22]. Both the native and invasive species are adapted to these free-draining volcanic ash soils of low fertility. In addition, large temperature extremes and varying rainfall conditions prevail [23,24,25].

Few studies have reported the impact of invasive plants on the surrounding flora and fauna in this ecosystem. A previous study found that plant communities dominated by tussock and other grasses were particularly vulnerable to heather invasion due to the invasive plant’s ability to germinate in inter-tussock spaces, its rapid vegetative growth, and environmental factors such as infertile soils associated with these communities [20]. Another study [16] reported displacement of native vegetation in heather-invaded sites on the Central Plateau and the author found that specialised phytophagous arthropods were negatively affected by the invasion, possibly due to a reduction in food availability, habitat loss and increased abundance of predators. The response of arthropod communities to broom invasion on the Central Plateau is not well documented. However, studies in other parts of New Zealand report an increase in generalist phytophages in broom-invaded areas [26,27]. This suggests that both heather and broom could have different effects on the composition of arthropod assemblages in this area.

This study aimed to establish the effects of two invasive species (heather and broom) on arthropod assemblages on the Central Plateau, North Island, New Zealand. Three different sampling methods were used; pitfall traps, flight intercept traps, and a beating tray [28]. Samples were collected from ten sites in summer and autumn, where invasive plants were either present (invaded) or absent (non-invaded) and occurring with different combinations of neighbouring plants. We first explored the differences in diversity (Richness, Simpson’s index, and Shannon’s index) and community composition at the order level for each sampling method for weed-invaded and non-invaded sites. We then used only the samples collected by the beating tray method to explore further the effect of different plant pairings on arthropod diversity and community composition. We predicted that the impact of invasive species on arthropod communities would vary depending on the invader, plant combination, and season. This study provides updated information on arthropod community composition in the region and a better understanding of the impacts of invasive plants on arthropods that will assist conservation efforts.

## 2. Results

### 2.1. Arthropods at Weed-Invaded vs. Non-Invaded Sites

In summer, we collected 13,465 arthropods belonging to 11 orders (Table 1). Order richness (R) for arthropods caught by the beating tray method was significantly different between weed-invaded and non-invaded sites (Kruskal–Wallis; *X*^2^ = 24.90, *p* < 0.001). However, order richness was not significantly different for arthropods caught either by flight intercept traps or pitfall traps (Table S1). Shannon’s and Simpson’s diversity indices did not differ between the sites for arthropods caught by the beating tray method, flight intercept, or pitfall traps (Table S1).

In autumn, we found lower numbers of arthropods, with 6,010 total individuals belonging to 11 orders caught in this season (Table 2). Again, order richness for arthropods collected by the beating tray method differed significantly between the sites (Kruskal–Wallis; *X*^2^ = 18.80, *p* < 0.001), while that from the flight intercept and pitfall traps did not (Table S1). Unlike summer, the Shannon’s and Simpson’s diversity indices differed significantly between the weed-invaded and non-invaded sites for the beating tray method (Shannon’s H: *X*^2^ = 12.82, *p* = 0.002 and Simpson’s D: *X*^2^ = 8.38, *p* = 0.015) but not for the flight intercept and pitfall traps (Table S1).

**Figure 1:**
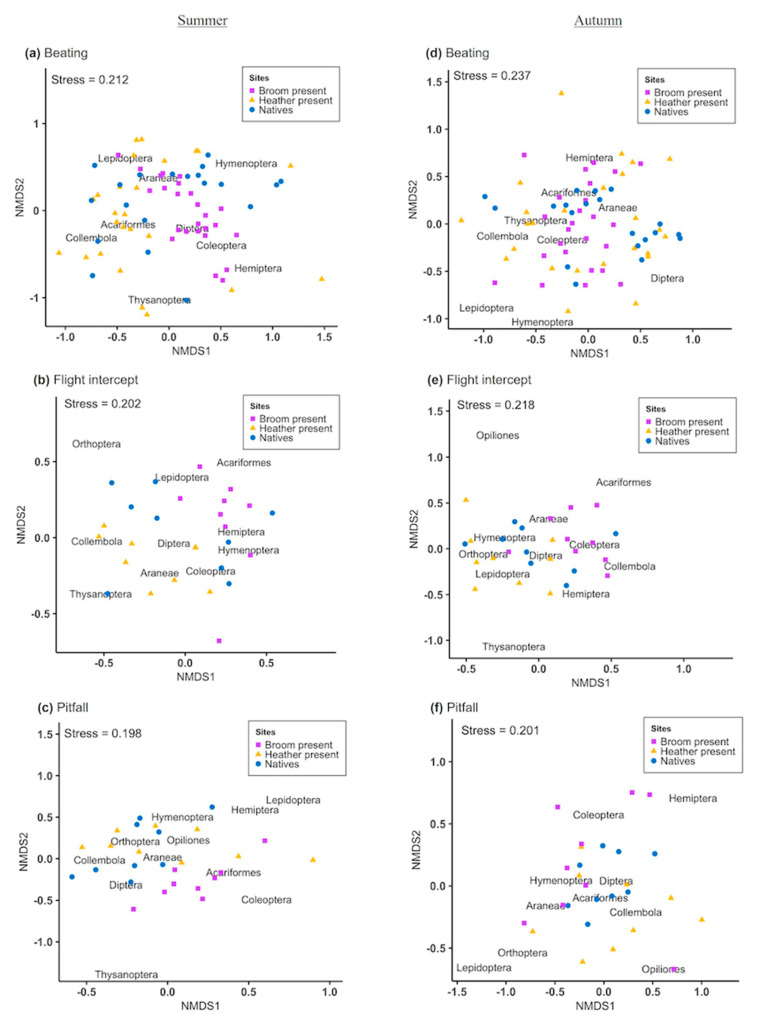
Non-metric multidimensional scaling (NMDS) plots for arthropod community composition in weed-invaded and non-invaded sites in summer (**a**–**c**) and autumn (**d**–**f**). Arthropods were caught by beating tray method, flight intercept, and pitfall traps.

A permutational analysis of variance (PERMANOVA) revealed that arthropod composition (relative abundance of insects belonging to each order) differed significantly between weed-invaded and non-invaded sites for all trapping methods in both seasons (Table 3). Differences in arthopod community composition between treatments for each sampling method can be visualized using non-metric multidimensional scaling (NMDS) plots (Figure 1).

The similarity percentage analyses revealed that different arthropod orders contributed to these differences depending on the season and trapping method used (Figure 1). The beating tray method data showed a high overlap among treatments in both summer and autumn. However, with flight intercept and pitfall traps, a higher separation between treatments was evident, with little overlap between the samples collected in heather-invaded and broom-invaded sites (Figure 1).

### 2.2. Arthropods Present on Target Plants under Different Plant Species Combinations

To explore the effect of different plant species combinations on arthropod communities, we only analysed the samples collected by the beating tray method on each target plant (heather, broom, manuka, or *Dracophyllum*) and classified samples according to the predominant plant combination present at each site (e.g., broom with mānuka, broom with *Dracophylum*, broom with heather, broom with conspecifics, and so forth). Likelihood ratio tests revealed arthropod orders to be affected differently depending on the plant combination and season (Table 4 and Table 5).

The Simpson diversity index was not affected by the neighbouring plant composition in all cases, except for broom in summer. With broom as the target plant, the Shannon diversity index was significantly different between sites where this plant co-occurred with either conspecifics or heterospecifics in both seasons. The Shannon diversity index for arthropods on mānuka only differed significantly between the sites in autumn. Order richness (R) was found to differ between plant combinations sharing the same target plant in at least one of the two seasons (Table 6).

A PERMANOVA revealed significant differences in arthropod order-level community composition on all target plants under different plant species combination (i.e., target plants paired with either conspecifics or one of the three heterospecific plants) during both seasons, except for broom in autumn (Table 7). 

**Figure 2:**
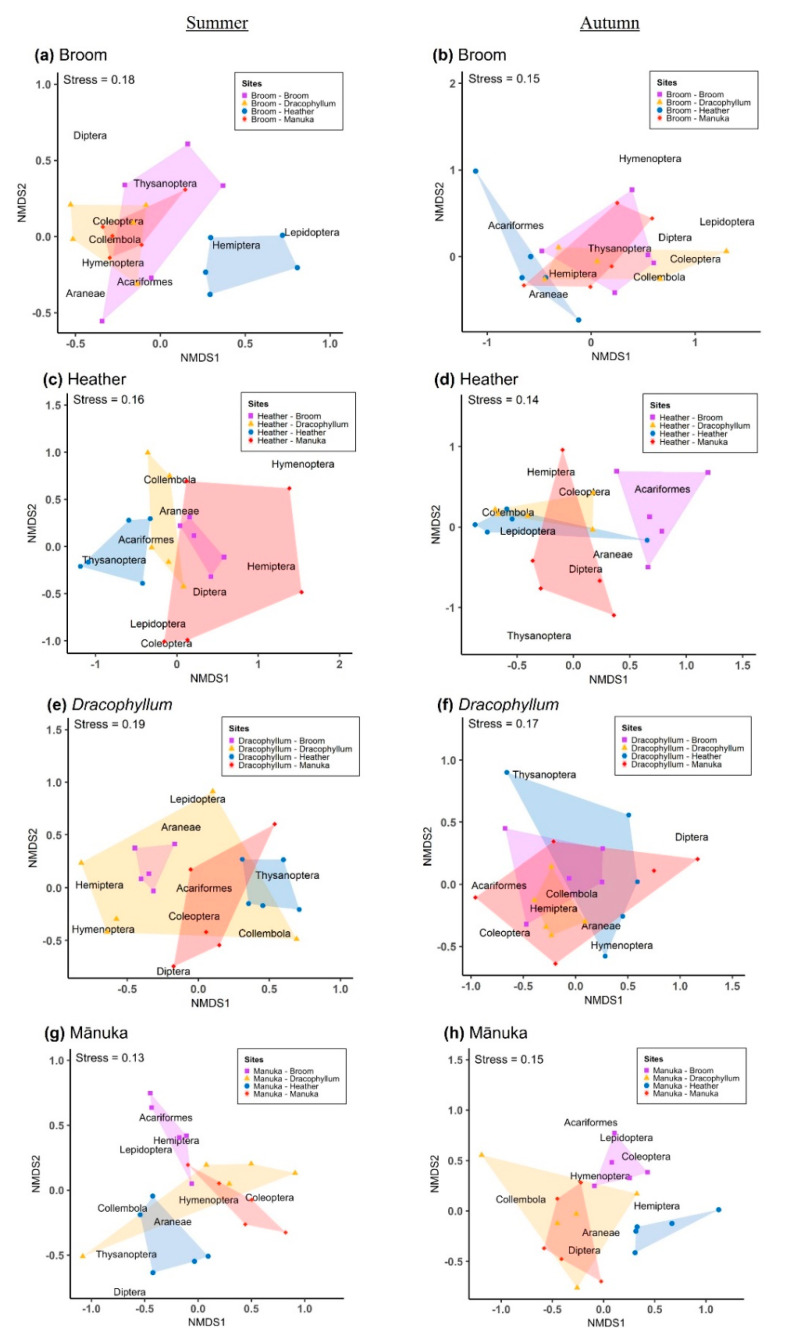
Non-metric multidimensional scaling (NMDS) plots showing the arthropod community composition on (**a,b**) broom, (**c,d**) heather, (**e,f**) *Dracophyllum,* and (**g,h**) mānuka paired with different plants in summer and autumn. Arthropods collected by the beating tray method (*n* = 5 for each group).

NMDS plots of the community composition (Figure 2), show that in the case of heather and broom, the arthropod composition has little overlap when plants are paired with conspecifics vs. when paired with another invasive both in summer and autumn. A similar trend was observed for mānuka when paired with conspecifics vs. either of the invasive plants, but this trend was not observed for *Dracophyllum*. Consistent with these observations, pairwise comparisons showed significant differences between treatments sharing the same target plant in both seasons, with very few exceptions (Table S2).

## 3. Discussion

Exotic plant invasion modifies vegetation structure and leads to a shift in plant species composition in the new habitat [29]. This may be detrimental for other community groups like arthropods that rely on surrounding native vegetation for food, shelter, and reproduction sites. Our results demonstrate that during plant invasion, arthropod assemblages are affected differently depending on the invading species. Broom typically increased arthropod abundance, while heather was associated with a reduction in arthropod abundance. Plant-arthropod associations were also affected by the identity of neighbouring plants, and these effects varied in summer and autumn.

Our results only partially support the often-reported observation that arthropod abundance and diversity are decreased in habitats dominated by exotic weeds [15], but rather indicate that the responses of arthropod communities depend on the identity of the invasive plant. While invasive plants may indeed reduce resources for specialist herbivores, arthropod groups occupying other, more generalist, ecological niches may thrive in these environments [15].

In this study, we found a high number of Acariformes (mostly detritivore oribatid mites) and Coleoptera (mostly silken fungus beetles) associated with broom in pitfall traps, while only small numbers of these groups were associated with heather. This is an example of how different invasive plants can provide different resources. Here, decaying vegetation under broom is creating optimal habitats for detritivores and fungivores [30,31].

In the flight intercept traps, Hemiptera, Coleoptera, Araneae, Acariformes, and Diptera were significantly more abundant in broom-infested sites in summer and autumn. Meanwhile, heather had less pronounced effects with only a substantial increase in Thysanoptera (thrips) and Araneae in summer and Orthoptera during autumn. Thrips are well-known generalist florivores and have been suggested to contribute to heather pollination in other ecosystems [32]. Many invasives have large floral displays or many flowers that can attract generalist pollinators and florivores [33]. It is, therefore, reasonable to assume that heather attracts native florivores which aid in its reproduction and dispersal, but no hard evidence was found that Hymenoptera (in particular native pollinators) captured by this method were significantly impacted by the presence of heather or broom.

The high numbers of Hemiptera found at the broom-invaded sites were predominantly exotic broom psyllids that were introduced from England to control the spread of broom in New Zealand [34]. In comparison, Coleoptera and Orthoptera were predominantly native generalist herbivores that would likely use invasive plants as an alternative food source. Araneae and some Diptera are predators or parasitoids of herbivores; thus, their increase may be explained by higher abundance of their prey species [35]. Certain plant architectures, such as the intricate and dense branching pattern of heather, can also create suitable habitats for spiders and other predators [16,36].

The composition of arthropods collected by the beating tray method showed a similar trend as that of flight intercept traps at broom-invaded sites but with higher abundance of Hemiptera, Coleoptera, Thysanoptera, and Acariformes. Contrary to this, a reduction in the abundance of some arthropod groups (Coleoptera, Diptera, Thysanoptera, and Orthoptera) was observed at the heather-invaded sites in samples collected by the beating tray method compared with the flight intercept traps. It is relevant to note that many Coleoptera caught by beating the foliage of mānuka were mānuka beetles, which are endemic insects typically associated with this plant. However, mānuka beetles have been reported to feed on other plants, and they are considered to be pasture pests in some regions [37], supporting our previous observation that some native insects feed on both native and exotic invasive plants [38].

An earlier study assessing the impact of invasion by heather on native invertebrates on the Central Plateau also showed some variation in invertebrate assemblages [16]. Consistent with our findings, the author found that invasion by heather was usually associated with fewer plant-feeders, high abundance of thrips (pollen eaters), and increased predators [16]. A comparative study of the arthropods associated with broom in two native (France and Scotland) and non-native (New Zealand and Australia) ranges [27], using the beating tray method, found generalist phytophages to be dominant on broom in exotic habitats and specialists dominant on broom in the native habitats. Thus, the overall abundance of arthropods was high but not significantly different between the two habitats. We also found high arthropod abundance in broom-invaded sites for several groups, suggesting that not only generalist herbivores, but arthropods occupying other niches, benefit from the resources provided by this invasive species. Generally, mechanisms promoting such facilitative interaction between invasive plants and arthropods include habitat modification, diversification of food source, and availability of exploitable hosts [39].

Plant species composition can be used as a predictor of arthropod assemblages, as revealed by a study using multiple sites with different levels of vegetation cover and a range of sampling methods [40]. However, that study revealed that this is not necessarily due to the direct use of particular plants as a resource, but their correlation with some other factors (i.e., microclimate, habitat structure, changes in trophic webs). Our results strongly support the hypothesis that it is plant community composition, rather than the presence of invaders only, that is a strong driver of change in arthropod assemblages. We found significant differences in arthropod community composition between sites where the same invasive was present but in combination with other species, and the same was true for native plants.

Overall, our results highlight that it is difficult to generalise when considering the impacts of invasive plants on arthropod communities, and that sampling multiple sites with different plant assemblages, using a variety of different trapping methods over multiple seasons, is needed to elucidate the complex effects of invasive plants on arthropod communities and their associated ecosystem services. Further studies investigating lower taxonomic levels, focusing on native and endemic arthropods and with a multitrophic approach [41], will be of great assistance to expand these findings and support conservation efforts.

## 4. Materials and Methods

### 4.1. Site Description

This study was conducted during summer 2017 through to autumn 2018 on the Central Plateau of the North Island, New Zealand. All of the woody plants used in the study occurred in natural and distinct combinations, creating an ideal system to characterise native–invasive plant interactions. We selected ten distinct sites where the four target woody shrub species—two natives, *Dracophyllum* and manuka, and two invasives, heather and broom—co-occur in all possible pairwise combinations (Supplementary Table S3). Five replicates of paired plants, either conspecific or heterospecific, of similar sizes, were selected at each site as target plants.

### 4.2. Arthropod Sampling Method

Three pitfall traps (76 mm deep × 90 mm diameter) covered with metal plates were laid between paired plants. Three flight intercept traps (220 mm × 500 mm) were positioned randomly about 50 cm off the ground at each site. A 50% propylene glycol solution was used as a preservative in both traps, and samples were collected after ten days. In addition, arthropods on all target plants were collected by beating similar portions of foliage from each plant onto a plastic tray. Arthropods caught in all traps were preserved in 70% ethanol and later identified to order. Sampling was first done in summer and repeated in autumn using the same techniques. The beating was done on the same target plants, and pitfall and flight intercept traps were positioned at the same locations during both seasons.

### 4.3. Data Analysis

All statistical analyses were performed using R (version 3.6.3). Firstly, we explored the effect of the presence of the two exotic invasive plants on arthropod composition using the three different sampling techniques. This was done by arranging samples into three categories: heather present (*n* = 25), broom present (*n* = 25), and natives only (*n* = 20). The site where both invasives were simultaneously present was excluded from the analyses. Comparisons were made for each sampling method.

To investigate the impact of neighbouring plants on arthropod order richness, diversity, and community composition, we used only data collected with the beating tray method. This allowed us to identify 16 separate treatments including all possible pairings between the four plants species, with five samples for each pair. Order richness and Shannon’s and Simpson’s diversity indices were calculated, and each variable was compared between treatments using the Kruskal–Wallis test. The abundance of arthropods was compared between sites using a negative binomial generalised linear model with site as the predictor and arthropod groups as the response variables. The significance of predictors was assessed using the likelihood ratio test.

Variations in arthropod community composition were assessed by permutational multivariate analysis (PERMANOVA) based on Bray–Curtis distance using the “adonis” function in the “vegan” package [42]. When PERMANOVA results were significant, the “pairwise.adonis” function was used to conduct pairwise analyses between sites. The similarity percentage analysis (SIMPER) was then used to identify the arthropod groups that contributed to the differences between sites. Non-metric multidimensional scaling (NMDS), also with Bray–Curtis distance, was used to visualise the changes in arthropod community composition. PERMANOVA and NMDS were both performed using square-root-transformed data [43,44].

## 5. Conclusions

We assessed arthropod communities in the Tongariro National Park (New Zealand) at sites where two exotic invasive weeds (heather and broom) were present or absent and investigated arthropod abundance on target plants during an invasion. Our work demonstrates that arthropod community composition in response to plant invasion is dependent on the identity of the invasive species and the composition of the nearby vegetation and shows that while some exotic invasive plants may reduce arthropod abundance and diversity in the new habitat, others may have the opposite effect. We also show that some of these effects may be seasonal and that results may vary depending on the sampling method used. This work emphasises the need for incorporating plant community composition, seasonality, and diverse sampling methods in future studies aimed at assessing the impact of invasive plants on arthropod communities.

## Figures and Tables

**Table 1:** The most abundant arthropod orders found at weed-invaded and non-invaded sites in summer. Comparisons between sites were performed using a negative binomial generalised linear model. Site was the predictor, while arthropod groups were used as the response variables. The likelihood ratio test was used to assess the significance of the predictors.

	Abundance (Mean ± SE)										
Site Per Trap	Collembola	Araneae	Hemiptera	Coleoptera	Hymenoptera	Diptera	Thysanoptera	Lepidoptera	Acariformes	Orthoptera	Opiliones
Beating tray											
Broom present	0.6 ± 0.5	3.4 ± 0.5	121.1 ± 33.3	59.1± 16.9	1.5 ± 0.3	0.9 ± 0.3	33.9 ± 10.9	0.6 ± 0.2	26.2 ± 6.6	ND	ND
Heather present	1.8 ± 0.6	1.6 ± 0.4	0.5 ± 0.2	0.9 ± 0.2	0.3 ± 0.1	0.9 ± 0.1	7.2 ± 2.6	0.4 ± 0.2	7.2 ± 2.0	ND	ND
Natives	1.4 ± 0.6	1.5 ± 0.3	1.2 ± 0.6	9.4 ± 3.1	1.5 ± 0.4	0.1 ± 0.1	0.2 ± 0.1	0.4 ± 0.2	4.1 ± 1.3	ND	ND
*X* ^2^	2.6	11.2	58.1	50.2	17.4	15.0	29.5	1.1	17.7	-	-
*p*-value	0.274	0.004 *	<0.001 *	<0.001 *	<0.001 *	0.001 *	<0.001 *	0.571	<0.001 *	-	-
Flight intercept											
Broom present	0.4 ± 0.2	1.3 ± 0.6	12.1 ± 2.3	18.3 ± 3.7	1.8 ± 0.4	18.3 ± 3.9	0.3 ± 0.2	1.7 ± 0.4	10.0 ± 3.1	0.2 ± 0.2	ND
Heather present	1.7 ± 0.5	3.9 ± 1.0	0.8 ± 0.2	5.8 ± 0.9	1.2 ± 0.3	14.1 ± 3.4	45.9 ± 20.2	0.9 ± 0.4	0.3 ± 0.2	0.7 ± 0.3	ND
Natives	0.4 ± 0.2	3.0 ± 0.6	1.2 ± 0.7	8.0 ± 2.4	1.4 ± 0.5	7.1 ± 1.2	0.4 ± 0.3	1.8 ± 0.5	1.0 ± 0.3	0.7 ± 0.6	ND
*X* ^2^	7.5	7.0	28.5	14.9	0.9	8.9	27.7	3.2	20.6	1.4	-
*p*-value	0.024 *	0.030 *	<0.001 *	0.001 *	0.624	0.012 *	<0.001 *	0.205	<0.001 *	0.494	-
Pitfall trap											
Broom present	3.7 ± 1.3	4.7 ± 0.6	0.3 ± 0.2	7.2 ± 1.4	6.6 ± 4.0	6.6 ± 1.2	0.4 ± 0.3	ND	20.3 ± 9.7	0.1 ± 0.1	0.4 ± 0.2
Heather present	8.4 ± 1.9	3.0 ± 0.7	0.1 ± 0.1	4.0 ± 2.5	15.2 ± 5.0	3.0 ± 2.6	ND	0.1 ± 0.1	2.2 ± 0.9	0.1 ± 0.1	0.8 ± 0.4
Natives	14.6 ±4.2	3.3 ± 1.0	0.4 ± 0.2	0.8 ± 0.8	13.2 ± 5.0	6.3 ± 1.5	ND	0.1 ± 0.1	1.0 ± 0.4	0.3 ± 0.2	0.6 ± 0.2
*X* ^2^	8.6	2.3	2.0	6.8	2.4	2.4	3.3	1.6	15.5	1.5	0.7
*p*-value	0.014 *	0.277	0.370	0.034 *	0.309	0.308	0.195	0.444	<0.001 *	0.476	0.694

Asterisks indicate significant differences between treatments for different orders and trapping methods (*p* < 0.005). ND = not detected.

**Table 2:** The most abundant arthropod orders found at weed-invaded and non-invaded sites in autumn. Comparisons between sites were performed using a negative binomial generalised linear model. Site was the predictor, while arthropod groups were used as the response variables. The likelihood ratio test was used to assess the significance of the predictors.

	Abundance (Mean ± SE)										
Site Per Trap	Collembola	Araneae	Hemiptera	Coleoptera	Hymenoptera	Diptera	Thysanoptera	Lepidoptera	Acariformes	Orthoptera	Opiliones
Beating tray											
Broom present	7.1 ± 3.1	5.5 ± 1.0	14.8 ± 5.3	9.4 ± 4.0	0.4 ± 0.1	0.7 ± 0.3	5.4 ± 3.2	0.4 ± 0.2	53.3 ± 17.8	ND	ND
Heather present	10.7 ± 3.1	3.2 ± 1.1	4.4 ± 2.0	0.4 ± 0.1	0.1 ± 0.1	0.6 ± 0.2	0.3 ± 0.2	0.3 ± 0.2	1.0 ± 0.5	ND	ND
Natives	3.0 ± 0.7	2.7 ± 0.5	1.9 ± 0.6	0.7 ± 0.2	0.2 ± 0.1	0.9 ± 0.3	0.2 ± 0.1	ND	2.2 ± 0.7	ND	ND
*X* ^2^	5.93	6.5	14.6	33.8	4.2	0.7	15.1	6.7	51.6	-	-
*p*-value	0.052	0.039 *	0.001 *	<0.001 *	0.122	0.697	0.001 *	0.035 *	<0.001 *	-	-
Flight intercept											
Broom present	33.3 ±11.8	33.3 ± 10.0	9.5 ± 5.3	35.0 ± 10.3	33.3 ± 8.8	40.6 ± 10.0	ND	16.7 ± 11.8	46.3 ± 14.9	11.1 ± 6.1	11.1 ± 11.1
Heather present	4.0 ± 2.2	12.5 ± 3.6	7.9 ± 5.4	5.4 ± 2.4	40.0 ± 10.0	35.6 ± 6.4	11.1 ± 11.1	11.1 ± 7.4	7.4 ± 7.4	44.4 ± 12.3	11.1 ± 11.1
Natives	25.3 ±11.1	12.5 ± 3.6	12.7 ± 11.0	7.4 ± 0.8	35.6 ± 11.4	31.8 ± 9.1	11.1 ± 11.1	27.8 ± 21.1	1.9 ± 1.9	19.4 ± 8.1	ND
*X* ^2^	7.8	7.1	0.2	18.1	0.2	0.6	1.6	1.4	48.3	6.6	1.6
*p*-value	0.021 *	0.029 *	0.920	<0.001 *	0.885	0.745	0.444	0.505	<0.001 *	0.038 *	0.444
Pitfall trap											
Broom present	5.8 ± 0.8	1.0 ± 0.3	0.2 ± 0.2	2.1 ± 0.8	1.9 ± 0.7	2.4 ± 1.5	ND	0.1 ± 0.1	8.3 ± 3.0	0.1 ± 0.1	0.9 ± 0.5
Heather present	19.9 ± 8.5	1.4 ± 0.6	0.1 ± 0.1	1.1 ± 0.6	4.2 ± 1.6	3.2 ± 1.5	ND	ND	3.8 ± 0.9	0.3 ± 0.2	1.3 ± 0.6
Natives	21.1 ± 5.0	1.3 ± 0.4	0.2 ± 0.2	1.2 ± 0.3	6.6 ± 2.7	7.2 ± 1.6	ND	ND	9.2 ± 6.0	0.1 ± 0.1	0.9 ± 0.5
*X* ^2^	10.7	0.5	0.4	1.6	4.1	3.4	-	2.2	2.7	1.2	6.1
*p*-value	0.004 *	0.772	0.840	0.442	0.129	0.184	-	0.333	0.263	0.550	0.048 *

Asterisks indicate significant differences between treatments for different orders and trapping methods (*p* < 0.005). ND = not detected.

**Table 3:** Statistical results for differences in community composition between treatments (broom present, heather present, and natives) after permutational analysis of variance (PERMANOVA), for three trapping methods.

	Summer		Autumn	
Trapping Method	Pseudo-*F*	*p*	Pseudo-*F*	*p*
Beating tray	*F*_2,67_ = 8.49	<0.001 *	*F*_2,67_ = 6.70	<0.001 *
Flight intercept	*F*_2,24_ = 7.75	<0.001 *	*F*_2,24_ = 3.06	0.003 *
Pitfall trap	*F*_2,24_ = 4.53	<0.001 *	*F*_2,24_ = 2.12	0.036 *

Asterisks indicate significant differences in community composition between treatments for each trapping method (*p* < 0.005).

**Table 4:** Arthropods on target plants when paired with either conspecific or heterospecific neighbours in summer. Arthropods were caught by beating a similar proportion of foliage of each target plant onto a tray (*n* = 5). Comparisons between sites were performed using a negative binomial generalised linear model. Site was used as the predictor, while arthropod groups were the response variables. The likelihood ratio test was used to assess the significance of the predictors.

Abundance (Mean ± SE)									
	Collembola	Araneae	Hemiptera	Coleoptera	Hymenoptera	Diptera	Thysanoptera	Lepidoptera	Acariformes
**Broom as target plant**									
Broom—Broom	ND	1.0 ± 0.6	103.2 ± 27.7	31.6 ± 8.7	1.4 ± 1.2	0.6 ± 0.4	71.0 ± 36.5	0.6 ± 0.4	18.8 ± 9.7
Broom—Heather	0.2 ± 0.2	1.2 ± 1.0	544.2 ± 95.9	2.2 ± 0.4	1.0 ± 0.8	ND	12.4 ± 5.0	2.2 ± 1.2	6.8 ± 2.0
Broom—*Dracophyllum*	2.2 ± 2.2	3.2 ± 0.7	310.8 ± 115.0	108.2 ± 16.1	1.2 ± 0.2	2.8 ± 1.2	23.2 ± 11.9	0.2 ± 0.2	5.8 ± 3.5
Broom—Mānuka	0.6 ± 0.6	4.8 ± 1.4	184.4 ± 47.3	151.4 ± 60.4	1.6 ± 0.6	0.8 ± 0.4	75.2 ± 26.7	0.2 ± 0.2	12.0 ± 4.2
*X* ^2^	25.6	8.4	14.4	38.2	0.4	12.3	7.1	7.6	3.7
*p*-value	<0.001 *	0.039 *	0.002 *	<0.001 *	0.932	0.006 *	0.070	0.054	0.296
***Dracophyllum* as target plant**									
*Dracophyllum*—*Dracophyllum*	0.8 ± 0.4	0.8 ± 0.6	3.4 ± 2.0	3.8 ± 1.6	1.2 ± 0.5	0.4 ± 0.2	0.2 ± 0.2	0.2 ± 0.2	ND
*Dracophyllum*—Heather	5.4 ± 2.2	1.2 ± 0.6	0.2 ± 0.2	2.0 ± 0.8	0.2 ± 0.2	ND	3.6 ± 1.1	ND	20.2 ± 3.5
*Dracophyllum*—Mānuka	4.4 ± 1.7	1.4 ± 0.7	0.8 ± 0.8	2.8 ± 1.4	0.6 ± 0.2	ND	0.4 ± 0.2	0.8 ± 0.6	9.2 ± 3.1
*Dracophyllum*—Broom	0.2 ± 0.2	5.6 ± 1.0	4.8 ± 1.5	2.6 ± 1.0	2.0 ± 0.6	0.2 ± 0.2	ND	0.4 ± 0.2	62.8 ± 25.1
*X* ^2^	13.6	14.2	8.6	0.9	54.0	4.5	19.3	4.9	41.1
*p*-value	0.004 *	0.003 *	0.035 *	0.819	<0.001 *	0.212	<0.001 *	0.180	<0.001 *
**Heather as target plant**									
Heather—Heather	1.0 ± 0.8	0.8 ± 0.6	1.0 ± 1.0	ND	ND	ND	21.2 ± 7.8	0.4 ± 0.4	11.6 ± 5.7
Heather—*Dracophyllum*	2.2 ± 0.5	2.0 ± 1.1	0.2 ± 0.2	0.8 ± 0.4	ND	0.2 ± 0.2	10.2 ± 7.8	ND	3.6 ± 1.2
Heather—Mānuka	0.2 ± 0.2	0.4 ± 0.2	1.2 ± 0.5	0.6 ± 0.4	0.2 ± 0.2	ND	0.4 ± 0.4	0.2 ± 0.2	ND
Heather—Broom	1.2 ± 0.6	1.6 ± 0.6	41.2 ± 12.1	0.2 ± 0.2	0.4 ± 0.4	0.6 ± 0.4	3.0 ± 1.1	0.2 ± 0.2	65.6 ± 20.1
*X* ^2^	7.5	4.9	26.2	6.5	3.3	5.8	13.9	2.3	33.5
*p*-value	0.059	0.179	<0.001 *	0.091	0.349	0.121	0.003 *	0.514	<0.001 *
**Mānuka as target plant**									
Mānuka—Mānuka	0.2 ± 0.2	2.2 ± 0.7	0.6 ± 0.2	10.6 ± 2.6	2.8 ± 1.4	0.2 ± 0.2	ND	0.2 ± 0.2	4.2 ± 2.9
Mānuka—Heather	ND	3.6 ± 0.9	ND	1.2 ± 0.4	1.0 ± 0.6	ND	0.4 ± 0.2	1.2 ± 0.6	0.6 ± 0.4
Mānuka—*Dracophyllum*	0.2 ± 0.2	1.6 ± 0.4	ND	20.4 ± 11.3	1.2 ± 0.8	ND	0.2 ± 0.2	0.2 ± 0.2	2.8 ± 1.1
Mānuka—Broom	0.2 ± 0.2	2.4 ± 0.5	2.4 ± 1.4	1.8 ± 0.8	1.2 ± 0.4	0.2 ± 0.2	0.2 ± 0.2	1.6 ± 0.8	31.8 ± 6.5
*X* ^2^	1.7	4.2	12.7	15.9	3.2	2.8	2.8	6.5	19.6
*p*-value	0.631	0.243	0.005 *	0.001 *	0.357	0.428	0.428	0.091	<0.001 *

Asterisks indicate significant differences between plant pairings having the same target plant for different orders (*p* < 0.005). ND = not detected.

**Table 5:** Arthropods on target plants when paired with either conspecific or heterospecific neighbours in autumn. Arthropods were caught by beating a similar proportion of foliage of each target plant onto a tray (*n* = 5). Comparisons between sites were performed using a negative binomial generalised linear model. Site was used as the predictor, while arthropod groups were the response variables. The likelihood ratio test was used to assess the significance of the predictors.

Abundance (Mean ± SE)									
	Collembola	Araneae	Hemiptera	Coleoptera	Hymenoptera	Diptera	Thysanoptera	Lepidoptera	Acariformes
**Broom as target plant**									
Broom—Broom	7.6 ± 4.5	10.2 ±3.1	39.2 ± 23.6	18.0 ± 13.3	0.2 ± 0.2	0.4 ± 0.4	17.0 ± 15.8	1.0 ± 0.8	132.8 ± 70.3
Broom—Heather	0.2 ± 0.2	2.2 ± 0.9	0.8 ± 0.8	ND	ND	ND	0.2 ± 0.2	ND	18.6 ± 13.0
Broom—*Dracophyllum*	1.4 ± 0.8	4.2 ± 3.0	6.4 ± 5.9	3.8 ± 1.9	ND	0.2 ± 0.2	1.2 ± 0.8	0.2 ± 0.2	15.0 ± 9.0
Broom—Mānuka	5.0 ± 3.8	4.8 ± 0.9	7.0 ± 2.8	1.0 ± 0.5	0.4 ± 0.2	0.2 ± 0.2	1.2 ± 0.8	ND	14.4 ± 6.9
*X* ^2^	7.1	6.6	8.8	14.7	4.5	2.3	6.5	6.0	11.8
*p*-value	0.070	0.086	0.032 *	0.002 *	0.212	0.514	0.091	0.112	0.008 *
***Dracophyllum* as target plant**									
*Dracophyllum*—*Dracophyllum*	6.0 ± 2.1	2.0 ± 0.6	4.0 ± 1.4	1.2 ± 0.6	0.4 ± 0.4	ND	0.4 ± 0.2	ND	4.8 ± 1.5
Dracophyllum—Heather	3.8 ± 1.5	8.4 ± 4.9	1.2 ± 0.4	0.2 ± 0.2	0.4 ± 0.2	1.0 ± 0.5	1.0 ± 0.6	ND	1.0 ± 0.6
*Dracophyllum*—Mānuka	2.2 ± 0.8	1.6 ± 0.7	ND	0.6 ± 0.4	ND	0.4 ± 0.2	0.2 ± 0.2	ND	0.6 ± 0.2
*Dracophyllum*—Broom	19.2 ± 14.1	3.6 ± 1.6	9.6 ± 6.5	18.0 ± 15.1	0.8 ± 0.4	0.8 ± 0.4	7.6 ± 3.7	ND	81.0 ± 39.9
*X* ^2^	9.3	6.9	17.2	15.2	5.4	7.7	13.8	-	31.4
*p*-value	0.026 *	0.075	0.001 *	0.002 *	0.144	0.053	0.003 *	-	<0.001 *
**Heather as target plant**									
Heather—Heather	29.8 ± 8.9	1.4 ± 0.4	1.2 ± 1.0	0.6 ± 0.2	ND	0.8 ± 0.4	ND	1.4 ± 0.7	1.0 ± 1.0
Heather—*Dracophyllum*	18.6 ± 6.6	1.6 ± 0.6	1.4 ± 0.8	0.6 ± 0.4	ND	ND	ND	0.2 ± 0.2	3.0 ± 2.3
Heather—Mānuka	1.6 ± 1.1	1.4 ± 0.7	0.2 ± 0.2	0.2 ± 0.2	ND	0.8 ± 0.4	0.6 ± 0.6	ND	0.2 ± 0.2
Heather—Broom	0.4 ± 0.4	2.4 ± 1.4	1.0 ± 1.0	2.0 ± 1.8	ND	0.2 ± 0.2	ND	ND	20.8 ± 9.7
*X* ^2^	17.6	1.2	2.0	3.8	-	7.6	0.3	10.0	12.5
*p*-value	0.001 *	0.750	0.579	0.284	-	0.055	0.963	0.018 *	0.006 *
**Mānuka as target plant**									
Mānuka—Mānuka	1.8 ± 0.9	3.8 ± 0.5	1.2 ± 0.7	0.6 ± 0.6	0.2 ± 0.2	1.2 ± 0.6	ND	ND	2.0 ± 2.0
Mānuka—Heather	ND	3.4 ± 1.3	18.2 ± 7.7	0.2 ± 02	ND	0.2 ± 0.2	ND	ND	ND
Mānuka—*Dracophyllum*	1.8 ± 0.8	3.4 ± 1.4	2.2 ± 1.5	0.4 ± 0.4	ND	1.8 ± 0.8	ND	ND	1.2 ± 0.7
Mānuka—Broom	2.4 ± 1.3	5.0 ± 1.1	12.0 ± 3.2	6.2 ± 2.5	0.4 ± 0.4	2.2 ± 1.5	ND	0.8 ± 0.6	23.4 ± 12.4
*X* ^2^	9.8	1.4	15.03	9.5	3.3	4.9	-	6.4	15.4
*p*-value	0.020 *	0.717	0.002 *	0.024 *	0.349	0.180	-	0.093	0.002 *

Asterisks indicate significant differences between plant pairings having the same target plant for different orders (*p* < 0.005). ND = not detected.

**Table 6:** Richness, Shannon’s and Simpson’s diversity indices for arthropods (at order level) on each target plant paired with conspecific and heterospecific neighbours in summer and autumn (*n* = 5). *p*-values were calculated using the Kruskal–Wallis test.

	Sites				Differences		
Target Plants	(Mean ± SE)				*X* ^2^	DF	*p*
**Summer**							
**Broom**	BB	BH	BD	BM			
Richness	5.8 ± 0.7	5.6 ± 0.2	6.8 ± 0.6	6.6 ± 0.5	3.5	3	0.319
Shannon	1.2 ± 0.2	0.3 ± 0.1	0.9 ± 0.1	1.1 ± 0.1	9.9	3	0.020 *
Simpson	0.6 ± 0.1	0.1 ± 0.0	0.5 ± 0.1	0.6 ± 0.1	10.8	3	0.013 *
Heather	HB	HH	HD	HM			
Richness	5.6 ± 0.2	3.2 ± 0.6	4.4 ± 0.9	2.4 ± 0.4	9.8	3	0.020
Shannon	1.0 ± 0.1	0.7 ± 0.2	1.1 ± 0.2	0.8 ± 0.2	2.1	3	0.549
Simpson	0.6 ± 0.0	0.4 ± 0.1	0.7 ± 0.1	0.7 ± 0.2	6.1	3	0.105
*Dracophyllum*	DB	DH	DD	DM			
Richness	5.4 ± 0.2	4.6 ± 0.0	3.2 ± 0.2	5.0 ± 0.9	8.3	3	0.041 *
Shannon	0.9 ± 0.1	1.1 ± 0.0	1.0 ± 0.1	1.3 ± 0.2	2.2	3	0.528
Simpson	0.4 ± 0.1	0.6 ± 0.0	0.7 ± 0.1	0.7 ± 0.1	3.0	3	0.061
Mānuka	MB	MH	MD	MM			
Richness	5.2 ± 0.6	3.8 ± 0.6	3.4 ± 0.5	4.40 ± 0.75	4.2	3	0.241
Shannon	0.9 ± 0.2	1.2 ± 0.1	0.8 ± 0.2	0.9 ± 0.2	3.6	3	0.303
Simpson	0.4 ± 0.1	0.8 ± 0.1	0.5 ± 0.1	0.5 ± 0.1	5.9	3	0.116
							
**Autumn**							
Broom	BB	BH	BD	BM			
Richness	5.6 ± 0.7	2.2 ± 0.4	4.4 ± 0.5	5.0 ± 0.9	9.4	3	0.024 *
Shannon	1.2 ± 0.2	0.5 ± 0.2	1.2 ± 0.1	1.1 ± 0.1	8.0	3	0.046 *
Simpson	0.6 ± 0.1	0.4 ± 0.1	0.8 ± 0.1	0.6 ± 0.0	7.4	3	0.060
Heather	HB	HH	HD	HM			
Richness	2.8 ± 0.7	4.0 ± 0.6	3.8 ± 0.4	2.8 ± 0.5	4.2	3	0.243
Shannon	0.6 ± 0.2	0.7 ± 0.2	0.7 ± 0.2	0.8 ± 0.2	0.7	3	0.884
Simpson	0.5 ± 0.2	0.3 ± 0.1	0.4 ± 0.1	0.8 ± 0.1	5.2	3	0.161
*Dracophyllum*	DB	DH	DD	DM			
Richness	6.4 ± 0.8	4.6 ± 0.8	5.2 ± 0.5	3.2 ± 0.4	9.1	3	0.028 *
Shannon	1.1 ± 0.1	1.2 ± 0.2	1.4 ± 0.1	1.0 ± 0.1	4.8	3	0.186
Simpson	0.6 ± 0.0	0.8 ± 0.1	0.8 ± 0.0	0.8 ± 0.1	5.7	3	0.125
Mānuka	MB	MH	MD	MM			
Richness	5.6 ± 0.2	2.2 ± 0.4	3.4 ± 0.7	3.4 ± 0.2	12.3	3	0.006 *
Shannon	1.4 ± 0.1	0.6 ± 0.2	1.0 ± 0.2	1.1 ± 0.1	10.0	3	0.018 *
Simpson	0.7± 0.1	0.4 ± 0.1	0.7 ± 0.0	0.7 ± 0.0	6.6	3	0.085

Asterisks indicate significant differences between treatments within the same row (*p* < 0.005). Abbreviations: Broom (B), heather (H), *Dracophyllum* (D), and mānuka (M). Combinations of abbreviations illustrate plant pairs, e.g., BB = broom paired with broom and BH = broom with heather.

**Table 7:** Permutational analysis of variance (PERMANOVA) results for differences in arthropod community composition on target plants at sites where they either occur with conspecifics or one of the three heterospecific plants (*n* = 5 for each treatment).

	Summer		Autumn	
Target plant	Pseudo-*F*	*P*	Pseudo-*F*	*p*
Broom	*F*_3,16_ = 5.12	<0.001*	*F*_3,16_ = 1.54	0.098
Heather	*F*_3,16_ = 6.12	<0.001*	*F*_3,16_ = 3.35	0.002 *
*Dracophyllum*	*F*_3,16_ = 4.30	<0.001*	*F*_3,16_ = 2.90	0.002 *
Mānuka	*F*_3,16_ = 3.71	0.002*	*F*_3,16_ = 3.38	0.002 *

Asterisks indicate significant differences between plant pairings sharing the same target plant (*p* < 0.005).

## Supplementary Materials

The following are available online at https://www.mdpi.com/2223-7747/9/7/919/s1, Figure S1: title, Table S1: title, Video S1: title.
