# Supplementary material for: Effects of Two Invasive Weeds on Arthropod Community Structure on the Central Plateau of New Zealand
Source: Plants (Basel). 2020 Jul 20;9(7):919. doi: 10.3390/plants9070919 (PMC7411625; doi:10.3390/plants9070919)
Supplement: Supplementary file 1 [file plants-09-00919-s001.pdf]

Supplementary material

Table S1. Order richness (R) and diversity indices for arthropods collected from sites where exotic invasive plants heather and broom were either present or absent.

Trap	Site (mean ± SE)			Test of differences	
	Broom present	Heather present	Natives	X^2	*P*-value
**Summer**					
**Beating**					
Order richness (R)	5.96 ± 0.26	3.72 ± 0.29	4.00 ± 0.34	24.90	< 0.001*
Shannon (H)	0.95 ± 0.07	0.97 ± 0.08	0.98 ± 0.09	0.09	0.954
Simpson (D)	0.49 ± 0.04	0.62 ± 0.05	0.58 ± 0.05	5.98	0.050
**Flight intercept**					
Order richness (R)	7.00 ± 0.47	7.33 ± 0.33	6.22 ± 0.32	4.81	0.090
Shannon (H)	1.45 ± 0.10	1.42 ± 0.11	1.56 ± 0.07	0.45	0.799
Simpson (D)	0.70 ± 0.05	0.68 ± 0.06	0.78 ± 0.03	2.85	0.241
**Pitfall**					
Order richness (R)	6.44 ± 0.24	5.22 ± 0.49	5.67 ± 0.41	4.81	0.090
Shannon (H)	1.51 ± 0.67	1.22 ± 0.12	1.25 ± 0.09	5.60	0.061
Simpson (D)	0.74 ± 0.03	0.64 ± 0.05	0.65 ± 0.04	2.88	0.237
**Autumn**					
**Beating**					
Order richness (R)	5.40 ± 0.31	3.48 ± 0.28	3.80 ± 0.29	18.80	< 0.001*
Shannon (H)	1.18 ± 0.05	0.77 ± 0.90	1.14 ± 0.07	12.82	0.002*
Simpson (D)	0.63 ± 0.03	0.53 ± 0.06	0.74 ± 0.03	8.38	0.015*
**Flight intercept**					
Order richness (R)	6.22 ± 0.40	4.78 ± 0.40	5.22 ± 0.55	4.89	0.087
Shannon (H)	1.41 ± 0.10	1.15 ± 0.10	1.27 ± 0.08	2.69	0.260
Simpson (D)	0.70 ± 0.04	0.62 ± 0.06	0.67 ± 0.04	058	0.750
**Pitfall**					
Order richness (R)	4.78 ± 0.40	5.11 ± 0.35	5.67 ± 0.33	2.46	0.293
Shannon (H)	1.18 ± 0.10	1.16 ± 0.15	1.21 ± 0.09	0.001	0.999
Simpson (D)	0.65 ± 0.04	0.59 ± 0.08	0.62 ± 0.05	0.149	0.928

Asterisks indicate significant differences between treatments for each trapping method (P < 0.005).

Table S2. Pairwise comparisons for arthropod community composition on target plants paired with conspecifics and heterospecific neighbours for both summer and autumn. Pairwise performed using the "pairwise.adonis" function in R.

	Test of differences				
**Sites/plant pairs**	**DF**	**Sum of Squares**	***F*-value**	R^2	*P*-value
**Summer**					
**Broom as target plant**					
Broom - Broom Vs. Broom - Heather	1	0.368	7.818	0.494	0.007*
Broom - Broom Vs. Broom - *Dracophyllum*	1	0.163	3.017	0.274	0.016*
Broom - Broom Vs. Broom - Mānuka	1	0.071	1.381	0.147	0.273
Broom - Heather Vs. Broom - *Dracophyllum*	1	0.306	9.154	0.534	0.007*
Broom - Heather Vs. Broom - Mānuka	1	0.355	11.344	0.586	0.007*
Broom - *Dracophyllum* Vs. Broom - Mānuka	1	0.048	1.250	0.135	0.310
***Dracophyllum* as target plant**					
Dracophyllum - *Dracophyllum* Vs. *Dracophyllum* - Heather	1	0.866	5.947	0.426	0.009*
Dracophyllum - *Dracophyllum* Vs. *Dracophyllum* - Mānuka	1	0.537	2.865	0.264	0.028*
Dracophyllum - *Dracophyllum* Vs. *Dracophyllum* - Broom	1	0.659	4.558	0.363	0.010*
Dracophyllum - Heather Vs. *Dracophyllum* - Mānuka	1	0.109	1.230	0.133	0.304
Dracophyllum - Heather Vs. *Dracophyllum* - Broom	1	0.446	9.850	0.552	0.008*
Dracophyllum - Mānuka Vs. *Dracophyllum* - Broom	1	0.389	4.468	0.358	0.016*
**Heather as target plant**					
Heather - Heather Vs. Heather - *Dracophyllum*	1	0.330	2.878	0.265	0.032*
Heather - Heather Vs. Heather - Mānuka	1	1.226	6.597	0.452	0.006*
Heather - Heather Vs. Heather - Broom	1	0.595	7.221	0.474	0.012*
Heather - *Dracophyllum* Vs. Heather - Mānuka	1	0.978	5.282	0.398	0.008*
Heather - *Dracophyllum* Vs. Heather - Broom	1	0.712	8.728	0.522	0.010*
Heather - *Mānuka* Vs. Heather - Broom	1	1.067	6.983	0.466	0.009*
**Mānuka as target plant**					
Mānuka - Mānuka Vs. Mānuka - Heather	1	0.395	4.239	0.346	0.022*
Mānuka - Mānuka Vs. Mānuka - *Dracophyllum*	1	0.135	0.991	0.110	0.461
Mānuka - Mānuka Vs. Mānuka - Broom	1	0.499	6.694	0.456	0.015*
Mānuka - Heather Vs. Mānuka - *Dracophyllum*	1	0.324	2.276	0.221	0.081
Mānuka - Heather Vs. Mānuka - Broom	1	0.565	6.998	0.467	0.008*
Mānuka - *Dracophyllum* Vs. Mānuka - Broom	1	0.495	3.997	0.333	0.010*
**Autumn**					
Dracophyllum as target plant					
Dracophyllum - *Dracophyllum* Vs. *Dracophyllum* - Heather	1	0.215	1.802	0.184	0.102
Dracophyllum - *Dracophyllum* Vs. *Dracophyllum* - Mānuka	1	0.348	3.037	0.275	0.019*
Dracophyllum - *Dracophyllum* Vs. *Dracophyllum* - Broom	1	0.331	3.689	0.316	0.023*
Dracophyllum - Heather Vs. *Dracophyllum* - Mānuka	1	0.258	1.405	0.149	0.302
Dracophyllum - Heather Vs. *Dracophyllum* - Broom	1	0.469	2.948	0.269	0.007*
Dracophyllum - Mānuka Vs. *Dracophyllum* - Broom	1	0.758	4.917	0.381	0.008*
**Heather as target plant**					
Heather - Heather Vs. Heather - *Dracophyllum*	1	0.152	1.252	0.135	0.269
Heather - Heather Vs. Heather - Mānuka	1	0.460	2.346	0.227	0.055
Heather - Heather Vs. Heather - Broom	1	0.977	6.586	0.451	0.027*
Heather - *Dracophyllum* Vs. Heather - Mānuka	1	0.381	2.138	0.211	0.060
Heather - *Dracophyllum* Vs. Heather - Broom	1	0.699	5.353	0.401	0.025*
Heather - Mānuka Vs. Heather - Broom	1	0.612	2.989	0.272	0.038*
**Mānuka as target plant**					

Mānuka - Mānuka Vs. Mānuka - Heather	1	0.590	5.364	0.401	0.011*
Mānuka - Mānuka Vs. Mānuka - *Dracophyllum*	1	0.041	0.245	0.030	0.912
Mānuka - Mānuka Vs. Mānuka - Broom	1	0.451	4.331	0.351	0.004*
Mānuka - Heather Vs. Mānuka - *Dracophyllum*	1	0.602	4.077	0.338	0.009*
Mānuka - Heather Vs. Mānuka - Broom	1	0.510	6.137	0.434	0.007*
Mānuka - *Dracophyllum* Vs. Mānuka - Broom	1	0.361	2.544	0.241	0.034*

Asterisks indicate significant differences between plant pairings ($P < 0.005$).Vs. = versus.

Table S3. Coordinates and plant species composition at study sites.

Site	Coordinates	Dominant plants	Description
1	Long. 175.685483 – Lat. -39.432933	Mānuka	Natives
2	Long. 175.685483 – Lat. -39.432933	Mānuka and *Dracophyllum*	Natives
3	Long. 175.68785 – Lat. - 39.4206	Heather and *Dracophyllum*	Heather present
4	Long. 175.73705 – Lat. -39.311217	*Dracophyllum*	Natives
5	Long. 175.734317 – Lat. -39.314683	Heather and Mānuka	Heather present
6	Long. 175.6888 – Lat. -39.415467	Heather	Heather present
7	Long. 175.737467 – Lat. -39.315117	Broom	Broom present
8	Long. 175.737 – Lat. -39.315383	Broom and Mānuka	Broom present
9	Long. 175.732783 – Lat. -39.3142	Broom and *Dracophyllum*	Broom present
10	Long. 175.3907 – Lat. -39.244033	Heather and Broom	Heather and broom present
